# Blowup Phenomena for the Compressible Euler and Euler-Poisson Equations with Initial Functional Conditions

**DOI:** 10.1155/2014/580871

**Published:** 2014-03-30

**Authors:** Sen Wong, Manwai Yuen

**Affiliations:** Department of Mathematics and Information Technology, The Hong Kong Institute of Education, 10 Lo Ping Road, Tai Po, New Territories, Hong Kong

## Abstract

We study, in the radial symmetric case, the finite time life span of the compressible Euler or Euler-Poisson equations in *R* ^*N*^. For time *t* ≥ 0, we can define a functional *H*(*t*) associated with the solution of the equations and some testing function *f*. When the pressure function *P* of the governing equations is of the form *P* = *Kρ* ^*γ*^, where *ρ* is the density function, *K* is a constant, and *γ* > 1, we can show that the nontrivial *C* ^1^ solutions with nonslip boundary condition will blow up in finite time if *H*(0) satisfies some initial functional conditions defined by the integrals of *f*. Examples of the testing functions include *r* ^*N*−1^ln(*r* + 1), *r* ^*N*−1^ *e* ^*r*^, *r* ^*N*−1^(*r* ^3^ − 3*r* ^2^ + 3*r* + *ε*), *r* ^*N*−1^sin((*π*/2)(*r*/*R*)), and *r* ^*N*−1^sinh *r*. The corresponding blowup result for the 1-dimensional nonradial symmetric case is also given.

## 1. Introduction

The compressible isentropic Euler (*δ* = 0) or Euler-Poisson (*δ* = ±1) equations for fluids can be written as

(1) $$\begin{array}{c} \rho_{t}+\nabla \cdot \left(\rho u\right)=0,\\ \rho \left[u_{t}+\left(u\cdot \nabla \right)u\right]+\nabla P=\rho \nabla \Phi ,\\ \Delta \Phi \left(t,x\right)=\delta \alpha \left(N\right)\rho , \end{array}$$

where *α*(*N*) is a constant related to the unit ball in *R* ^*N*^. As usual, *ρ* = *ρ*(*t*, *x*) ≥ 0 and *u* = *u*(*t*, *x*) ∈ **R** ^*N*^ are the density and the velocity, respectively. *P* = *P*(*ρ*) is the pressure function. The *γ*-law for the pressure term *P*(*ρ*) can be expressed as

(2) $$\begin{array}{c} P\left(\rho \right)=K\rho^{\gamma }, \end{array}$$

for which the constant *γ* ≥ 1. If *K* > 0, it is a system with pressure. If *K* = 0, it is a pressureless system.

When *δ* = −1, the system is self-attractive. The system (1) is the Newtonian description of gaseous stars (cf. [1, 2]). When *δ* = 1, the system comprises the Euler-Poisson equations with repulsive forces and can be applied as a semiconductor model [3]. When *δ* = 0, the system comprises the compressible Euler equations and can be applied as a classical model in fluid mechanics [4, 5].

The solutions in radial symmetry are expressed by

(3) $$\begin{array}{c} \rho =\rho \left(t,r\right),\;\;u=\frac{x}{r}V\left(t,r\right)=:\frac{x}{r}V, \end{array}$$

with the radius *r* = (∑_*i*=1_^*N*^ *x*_*i*_^2^)^1/2^.

The Poisson equation (1)_3_ becomes

(4) $$\begin{array}{c} \Phi_{r}\left(t,r\right)=\frac{\alpha \left(N\right)\delta }{r^{N-1}}\int_{0}^{r}\rho \left(t,s\right)s^{N-1}ds. \end{array}$$

The equations in radial symmetry can be expressed in the following form:

(5) $$\begin{array}{c} \rho_{t}+V\rho_{r}+\rho V_{r}+\frac{N-1}{r}\rho V=0,\\ \rho \left(V_{t}+VV_{r}\right)+P_{r}=\rho \Phi_{r}. \end{array}$$

The blowup phenomena have attracted the attention of many mathematicians. Regarding the Euler equations (*δ* = 0), Makino et al. [6] first investigated the blowup of “tame solutions.” In 1990, Makino and Perthame further analyzed the corresponding solutions for the equations with gravitational forces (*δ* = −1) [7]. Subsequently, Perthame [8] studied the blowup results for the 3-dimensional pressureless system with repulsive forces (*δ* = 1). Additional results of the Euler system can be found in [9–12].

In this paper, we introduce the nonslip boundary condition [13], which is expressed by

(6) $$\begin{array}{c} \rho \left(t,R\right)=0,\;\;V\left(t,R\right)=0, \end{array}$$

for all *t* ≥ 0 and with the constant *R* > 0.

In 2011, Yuen used the integration method to show the *C* ^1^ blowup phenomenon with a “radial dependent” initial functional:

(7) $$\begin{array}{c} I_{0}=\int_{0}^{R}r^{n}V_{0}dr>0, \end{array}$$

for *n* = 1 [14] and *n* > 0 [15].

Following the integration method, we observe that the functional (7) could be generalized to have the following result.

**Theorem 1**

Define the functional associated with the testing function *f* by

(8) $$\begin{array}{c} H\left(t\right)=\int_{0}^{R}f\left(r\right)V\left(t\right)dr \end{array}$$

and denote the initial functional *H*(0) by *H*_0_. Consider the Euler or Euler-Poisson equations (1) in *R* ^*N*^. For pressureless fluids (*K* = 0) or *γ* > 1, and the nontrivial classical *C* ^1^ solutions (*ρ*, *V*) with radial symmetry and the first boundary condition (6), we have the following results.

(a) For the attractive forces (*δ* = −1), if *H*_0_ satisfies the following initial functional condition:

(9) $$\begin{array}{c} \frac{H_{0}^{2}}{2R\int_{0}^{R}f\left(r\right)dr}-M\int_{0}^{R}\frac{f\left(r\right)}{r^{N-1}}dr>0, \end{array}$$

with a total mass *M* of the fluid and an arbitrary nonnegative and nonzero *C* ^1^[0, *R*] testing function *f*(*r*) satisfying the following properties:

1. lim⁡_*r*→0_(*f*(*r*)/*r* ^*N*−1^) exists,
2. *f*(*r*)/*r* is increasing,

then the solutions blow up in finite time.

(b) For the nonattractive forces (*δ* = 0 or 1), if *H*_0_ satisfies the following initial functional condition:

(10) $$\begin{array}{c} H_{0}=\int_{0}^{R}f\left(r\right)V_{0}dr>0, \end{array}$$

then the solutions blow up on or before the finite time *T* = (2*R*∫_0_^*R*^ *f*(*r*)*dr*)/*H*_0_.

## 2. The Generalized Integration Method

The key ideas in obtaining the above results are (i) to design the right form of generalized functional and find the right class of testing functions and (ii) to transform the nonlinear partial differential equations into the Riccati inequality.

**Proof**

The density function *ρ*(*t*, *x*(*t*; *x*)) conserves its nonnegative nature.

The mass equation (1)_1_

(11) $$\begin{array}{c} \frac{D\rho }{Dt}+\rho \nabla \cdot u=0, \end{array}$$

with the material derivative

(12) $$\begin{array}{c} \frac{D}{Dt}=\frac{\partial }{\partial t}+\left(u\cdot \nabla \right), \end{array}$$

could be integrated as

(13) ρ(t,x0) =ρ0(x0(0,x0))exp⁡(−∫0t∇·u(t,x0(t;x0))dt)≥0

for *ρ*_0_(*x*_0_(0, *x*_0_)) ≥ 0.

For the nontrivial density initial condition in radial symmetry, *ρ*_0_(*r*) ≠ 0, we have

(14) $$\begin{array}{c} V_{t}+VV_{r}+K\gamma \rho^{\gamma -2}\rho_{r}=\Phi_{r}\\ V_{t}+\frac{\partial }{\partial r}\left(\frac{1}{2}V^{2}\right)+K\gamma \rho^{\gamma -2}\rho_{r}=\Phi_{r}\\ f\left(r\right)V_{t}+f\left(r\right)\frac{\partial }{\partial r}\left(\frac{1}{2}V^{2}\right)+K\gamma f\left(r\right)\rho^{\gamma -2}\rho_{r}=f\left(r\right)\Phi_{r}. \end{array}$$

(Here we multiplied the function *f*(*r*) on both sides.)

Subsequently, we take integration with respect to *r* from 0 to *R* for *γ* > 1 or *K* = 0:

(15) ∫0Rf(r)Vtdr+∫0Rf(r)ddr(12V2)  +∫0RKγf(r)ργ−2ρrdr=∫0Rf(r)Φrdr.

(a) For *δ* = −1, we have

(16) ∫0Rf(r)Vtdr+∫0Rf(r)ddr(12V2)  +∫0RKγf(r)γ−1dργ−1 =−∫0R[α(N)f(r)rN−1∫0rρ(t,s)sN−1ds]dr,∫0Rf(r)Vtdr+∫0Rf(r)ddr(12V2)  +∫0RKγf(r)γ−1dργ−1≥−∫0R[f(r)MrN−1]dr,

with the total mass

(17) $$\begin{array}{c} M=\alpha \left(N\right)\int_{0}^{R}\rho \left(t,s\right)s^{N-1}ds. \end{array}$$

Then we apply the integration by parts to deduce

(18) ∫0Rf(r)Vtdr−12∫0RV2df(r) +12[f(r)V2(t,r)]|r=0r=R−∫0RKγf′(r)γ−1ργ−1dr +Kγγ−1[f(r)ργ−1(t,r)]|r=0r=R≥−∫0R[f(r)MrN−1]dr.

Inequality (18) with the first boundary condition (6) becomes

(19) $$\begin{array}{c} \frac{d}{dt}\int_{0}^{R}VdF\left(r\right)\geq \frac{1}{2}\int_{0}^{R}V^{2}f^{\prime }\left(r\right)dr-\int_{0}^{R}\left[\frac{f\left(r\right)M}{r^{N-1}}\right]dr, \end{array}$$

with *dF*(*r*) = *f*(*r*)*dr* and *γ* > 1 or *K* = 0.

Note that *f*(0) = 0 by property 1 and *f* is increasing by property 2.

Now, we define the assistant functional:

(20) $$\begin{array}{c} H\left(t\right)=\int_{0}^{R}f\left(r\right)Vdr=\int_{0}^{R}VdF\left(r\right). \end{array}$$

We then use the Cauchy-Schwarz inequality to obtain

(21) $$\begin{array}{c} \left|\int_{0}^{R}V\cdot 1dF\left(r\right)\right|\leq {\left(\int_{0}^{R}V^{2}dF(r)\right)}^{1/2}{\left(\int_{0}^{R}1dF(r)\right)}^{1/2}\\ \left|\int_{0}^{R}V\cdot 1dF\left(r\right)\right|\leq {\left(\int_{0}^{R}V^{2}f(r)dr\right)}^{1/2}{\left(\int_{0}^{R}f(r)dr\right)}^{1/2}\\ 0\leq \frac{\left|\int_{0}^{R}VdF\left(r\right)\right|}{{\left(\int_{0}^{R}f(r)dr\right)}^{1/2}}\leq {\left(\int_{0}^{R}V^{2}f(r)dr\right)}^{1/2} \end{array}$$

for *R* > 0,

(22) $$\begin{array}{c} \frac{H^{2}\left(t\right)}{\int_{0}^{R}f\left(r\right)dr}\leq \int_{0}^{R}V^{2}f\left(r\right)dr, \end{array}$$

(23) $$\begin{array}{c} \frac{H^{2}\left(t\right)}{2R\int_{0}^{R}f\left(r\right)dr}\leq \frac{1}{2R}\int_{0}^{R}V^{2}f\left(r\right)dr. \end{array}$$

In view of (23) and (19), we get

(24) ddtH(t)≥12∫0RV2f′(r)dr−M∫0Rf(r)rN−1dr≥12R∫0RV2f(r)dr−M∫0Rf(r)rN−1dr≥H(t)22R∫0Rf(r)dr−M∫0Rf(r)rN−1dr,

(25) $$\begin{array}{c} \frac{d}{dt}H\left(t\right)\geq \frac{H{\left(t\right)}^{2}}{2R\int_{0}^{R}f\left(r\right)dr}-M\int_{0}^{R}\frac{f\left(r\right)}{r^{N-1}}dr, \end{array}$$

as *f*′(*r*)≥(1/*r*)*f*(*r*) by property 2.

It is well known that, with the initial condition

(26) $$\begin{array}{c} \frac{H_{0}^{2}}{2R\int_{0}^{R}f\left(r\right)dr}-M\int_{0}^{R}\frac{f\left(r\right)}{r^{N-1}}dr>0, \end{array}$$

the Riccati inequality (25) will blow up on or before the finite time *T*.

(b) For *δ* = 0 or 1, by a similar analysis, one can show that

(27) $$\begin{array}{c} \frac{d}{dt}H\left(t\right)\geq \frac{H{\left(t\right)}^{2}}{2R\int_{0}^{R}f\left(r\right)dr}. \end{array}$$

Finally,

(28) $$\begin{array}{c} H\left(t\right)\geq \frac{-H_{0}}{\left(H_{0}/\left(2R\int_{0}^{R}f\left(r\right)dr\right)\right)t-1}, \end{array}$$

if we set the initial condition

(29) $$\begin{array}{c} H_{0}=\int_{0}^{R}f\left(r\right)V_{0}dr>0. \end{array}$$

Thus, the solutions blow up on or before the finite time *T* = (2*R*∫_0_^*R*^ *f*(*r*)*dr*)/*H*_0_.

The proof is completed.

**Remark 2**

For the physical explanation of the functional *H*(*t*), readers may refer to Sideris' paper [16].

For the construction of testing functions *f* with the desired properties as required in Theorem 1, one recalls the class of power series:

(30) $$\begin{array}{c} \sum_{i=0}^{\infty }a_{i}x^{i}, \end{array}$$

with the following properties:

- all *a*_*i*_ ≥ 0 for all *i* and *a*_*i*_ = 0 for *i* < *N* − 1,
- the radius of convergence is not less than *R*.

Actually, power series (or real analytic functions) with the above properties constitute a large class of examples for *f*. Concrete examples include *r* ^*N*−1^ *e* ^*r*^ and *r* ^*N*−1^sinh⁡*r*. Moreover, there are examples with some *a*_*i*_ < 0: *r* ^*N*−1^ln⁡(*r* + 1), *r* ^*N*−1^sin((*π*/2)(*r*/*R*)), and *r* ^*N*−1^(*r* ^3^ − 3*r* ^2^ + 3*r* + *ɛ*), where the constant *ɛ* > 0 can be arbitrary.

## 3. The 1-Dimensional Nonradial Symmetric Case

In the 1-dimensional case, we can apply a similar argument to gain the result for the nonradial symmetric fluids.

**Theorem 3**

Suppose *u* and *ρ* have compact support on [*a*, *b*] and vanish at the boundaries:

(31) $$\begin{array}{c} u\left(t,a\right)=u\left(t,b\right)=\rho \left(t,a\right)=\rho \left(t,b\right)=0, \end{array}$$

for all *t* ≥ 0. By considering *u*(*t*, *x* − *a*) and *ρ*(*t*, *x* − *a*) instead, one may suppose *a* ≥ 0. Let *f*(*x*) be a nonnegative and nonzero *C* ^1^[*a*, *b*] testing function, such that *f*(*x*)/*x* is increasing for *x* > *a* and the functional is given by

(32) $$\begin{array}{c} H\left(t\right)=\int_{a}^{b}f\left(x\right)u\left(x,t\right)dx. \end{array}$$

(a) For *δ* = 1 or −1, if the initial functional *H*_0_ satisfies
  (33) $$\begin{array}{c} \frac{H_{0}^{2}}{2b\int_{a}^{b}f\left(x\right)dx}-\frac{M}{2}\int_{a}^{b}f\left(x\right)dx>0, \end{array}$$
 then the solutions blow up in finite time.
(b) For *δ* = 0, if *H*_0_ > 0, then the solutions blow up on or before the finite time *T* = (2*b*∫_*a*_^*b*^ *f*(*x*)*dx*)/*H*_0_.

**Proof**

For the 1-dimensional case, (1)_2_ becomes

(34) $$\begin{array}{c} u_{t}+uu_{x}+K\gamma \rho^{\gamma -2}\rho_{x}=\Phi_{x}. \end{array}$$

For *γ* ≠ 1, one has

(35) $$\begin{array}{c} u_{t}+\frac{1}{2}\frac{\partial u^{2}}{\partial x}+\frac{K\gamma }{\gamma -1}\frac{\partial \rho^{\gamma -1}}{\partial x}=\Phi_{x}. \end{array}$$

Then, we multiply the above equation by *f*(*x*) on both sides, taking integration with respect to *x* from *a* to *b* and using integration by parts, to yield

(36) ddtH(t)+12(f(x)u2)|x=bx=a−12∫abu2f′(x)dx  +Kγγ−1[(f(x)ργ−1)|x=bx=a−∫abργ−1f′(x)dx] =∫abf(x)Φxdx.

As *u*(*t*, *a*) = *u*(*t*, *b*) = *ρ*(*t*, *a*) = *ρ*(*t*, *b*) = 0, for all *t*, we get

(37) ddtH(t)=12∫abu2f′(x)dx +Kγγ−1∫abργ−1f′(x)dx+∫abf(x)Φxdx≥12∫abu2f′(x)dx+∫abf(x)Φxdx.

Using the properties of *f*(*x*) and the Cauchy-Schwarz inequality (as in the proof of Theorem 1), we obtain

(38) $$\begin{array}{c} \frac{d}{dt}H\left(t\right)\geq \frac{H^{2}\left(t\right)}{2b\int_{a}^{b}f\left(x\right)dx}+\int_{a}^{b}f\left(x\right)\Phi_{x}dx. \end{array}$$

On the other hand, by using the following explicit form of Φ_*x*_:

(39) $$\begin{array}{c} \Phi_{x}\left(t,x\right)=\frac{\delta }{2}\left(\int_{a}^{x}\rho \left(t,x\right)dy-\int_{x}^{b}\rho \left(t,x\right)dy\right) \end{array}$$

and the following estimate:

(40) $$\begin{array}{c} \Phi_{x}\geq -\frac{\left|\delta \right|}{2}M, \end{array}$$

we get the following.

(a) For *δ* = 1 or −1,

(41) $$\begin{array}{c} \frac{d}{dt}H\left(t\right)\geq \frac{H^{2}\left(t\right)}{2b\int_{a}^{b}f\left(x\right)dx}-\frac{M}{2}\int_{a}^{b}f\left(x\right)dx. \end{array}$$

(b) For *δ* = 0,

(42) $$\begin{array}{c} \frac{d}{dt}H\left(t\right)\geq \frac{H^{2}\left(t\right)}{2b\int_{a}^{b}f\left(x\right)dx}. \end{array}$$

Thus, the result immediately follows.
